# 

**Published:** 1852-03

**Authors:** 


### WESTERN JOURNAL

### OF

### MEDICINE AND SURGERY.

### EDITORS.

---

LUNSFORD P. YANDELL, M. D.
PROFESSOR OF PHYSIOLOGY AND PATHOLOGICAL ANATOMY IN THE UNIVERSITY OF LOUISVILLE.

AND

THEODORE S. BELL, M.D.

### RECEIPTS OF MEDICAL JOURNAL FOR FEB. 1852.

| | |
|---|---|
| Dr. Cole, Cataract, Ind, | $5 00 |
| J. M. Stucky, Gosport, Ind | 10 00 |
| W. H. Adair, Cynthiana, Ky | 10 00 |
| R. A. New, Rodney, Miss | 5 00 |
| S. W. McClurken, Halselville, S. C. | 5 00 |
| W. Brice, Youngsville, S. C | 5 00 |
| A. B. Palmer, Harrisonville, Mo | 5 00 |
| F. Cannon, Jackson, Mo | 5 00 |
| D. L. Freeman, Floydsburg, Ky | 5 00 |
| S. McCollough, Brownsboro, Ark | 5 00 |
| G. P. Robinson, office | 5 00 |
| B. D. Ragland, Wellington, Mo | 5 00 |
| J. H. Hutcherson, Keysburg, Ky | 5 00 |
| E. G. Harris, Fayette, Ala | 2 00 |
| E. Murphey, New Harmony, Ind | 5 00 |
| J. B. Holloway, Myers' Mills, Ill | 5 00 |
| T. Lipscomb, Shelbyville, Tenn | 5 00 |
| E. L Minor, Lithopolis, Ohio | 10 00 |
| J. J. Bailey, Allsboro, Ala | 5 00 |
| —— Davis, Jackson, Mo | 5 00 |
| W. S. Holland, Calhoon, Mo | 5 00 |
| J. Dunn, Warsaw, Mo | 5 00 |

**The Western Journal of Medicine and Surgery is published monthly by the undersigned, at the corner of Main and Fifth streets, Louisville, at $5 per annum, payable in advance. Each number contains 96 pages, making two volumes in the year of more than 550 pages each.**

**Contributions to its pages by the Physicians of the Valley of the Mississippi addressed to the Editors, are respectfully solicited.**

**Letters on business, to be addressed, postage paid, to the publishers.**

PRENTICE & HENDERSON.

